# Oral anticoagulant timing and hospitalization in newly diagnosed nonvalvular atrial fibrillation patients

**DOI:** 10.3389/fcvm.2025.1522154

**Published:** 2025-04-04

**Authors:** Chendi Cui, Laura Curry, Nisha Singh, Ning An Rosenthal

**Affiliations:** ^1^ Premier Applied Sciences, Premier Inc., Charlotte, NC, United States; ^2^ Bristol-Myers Squibb, Dallas-Fort Worth, TX, United States

**Keywords:** nonvalvular atrial fibrillation, oral anticoagulants, hospitalization, timing of initiation, real-world evidence

## Abstract

**Background:**

Non-valvular atrial fibrillation (NVAF) significantly increases ischemic stroke and systemic embolism (SE) risks. Despite the proven efficacy of oral anticoagulants (OAC) in reducing these risks, their underutilization highlights a gap in clinical practice. This study examined OAC utilization patterns within the first year after NVAF diagnosis in patients without prior OAC use and the association between the timing of OAC initiation and the risk of all-cause and stroke/SE-specific hospitalizations.

**Methods:**

A retrospective cohort study was conducted using data from the Premier Healthcare Database and linked claims from 1/1/2017–3/31/2021. Patients newly diagnosed with NVAF, without prior OAC use, were included.

**Results:**

Of 23,148 adults with newly diagnosed NVAF, 11,059 (47.8%) initiated OAC within one year. OAC users predominantly had cardiovascular disease and risk factors, whereas non-OAC users had higher rates of malignancy and dementia. Early OAC initiation (74.9% during the index visit) was linked to lower hospitalization risks compared to those initiating later (29.2% vs. 45.9% for all-cause, *p-value* < 0.001 and 1.3% vs. 2.6% for stroke/SE-specific, *p-value* < 0.001). Adjusted odds ratios for all-cause and stroke/SE hospitalization favored early initiation were 0.35 (95% CI: 0.32–0.39) and 0.34 (95% CI: 0.24–0.47), respectively.

**Conclusions:**

This study highlights OAC underutilization in NVAF patients and suggests early initiation may lower hospitalization rates. The findings emphasize the need for further research into real-world compliance with OAC guidelines and call for further research to confirm the benefits of early initiation. Personalized management strategies that consider individual patient profiles are recommended.

## 1 Introduction

Atrial fibrillation (AF) is the most common cardiac arrhythmia in clinical practice, significantly increasing the risk of mortality and thromboembolic events, especially ischemic stroke (IS) and systemic embolism (SE) (1, 2). Non-valvular atrial fibrillation (NVAF), defined as AF without mechanical prosthetic heart valves or moderate-to-severe mitral stenosis (3, 4), further amplifies stroke risk, accounting for 20%–30% of all IS worldwide (1, 5). Given the substantial individual and societal implications of stroke, comprehensive management of NVAF is a key component of IS prevention (6).

The United States (U.S.) and European guidelines recommend oral anticoagulants (OAC)—including vitamin K antagonists like Warfarin and direct oral anticoagulants (DOACs, such as dabigatran, rivaroxaban, apixaban, and edoxaban)—to reduce IS and SE risks in patients with NVAF. Although their efficacy and safety have been demonstrated by rigorous randomized clinical trials (RCTs), real-world implementation of these agents frequently encounters hurdles with their notable underutilization (7–10). While recent U.S. studies note an uptrend in OAC use, with 33%–65% of AF patients receiving OACs, substantial variations persist across different populations (11–13). This inconsistency underscores the need for further investigation into OAC utilization patterns among U.S. patients with NVAF.

Moreover, literature regarding the optimal timing for OAC initiation relative to stroke risk in NVAF patients is limited. Although individual studies have reported mixed results (14–17), a recent systematic review and meta-analysis on patients with acute ischemic stroke found that early OAC initiation was associated with a reduced risk of composite outcomes, including ischemic stroke recurrence, intracranial hemorrhage, major bleeding, and all-cause mortality (18). This correlation remains unexplored in patients without prior strokes. In addition, to our knowledge, no prior research has investigated this association in a real-world setting.

This study aimed to describe OAC usage patterns within the first-year post diagnosis of NVAF among patients with no prior OAC use. Additionally, we examined the relationship between the timing of OAC initiation and the one-year risk of all-cause and stroke/SE-specific hospitalizations for patients starting OAC within this timeframe.

## 2 Materials and methods

### 2.1 Design, data, and setting

This retrospective observational study used data from the Premier Healthcare Database (PHD) and its linked medical and pharmacy insurance claims database. The claims data and PHD data were linked via tokenization. As per US Title 45 Code of Federal Regulations, Part 46, this study of fully deidentified data was exempted from institutional review board approval, as with prior PHD studies (19–22). Researchers followed the Strengthening the Reporting of Observational Studies in Epidemiology (STROBE) checklist (Supplementary Table S1).

### 2.2 Study population

The index period spanned from January 1, 2017 through March 31, 2021 with a 365-day look-back and follow-up period (Supplementary Figure). Patients were included if they met all of the following criteria: (1) age ≥18 years, (2) had an inpatient visit with a principal or secondary discharge diagnosis of AF as defined by ICD-10-CM diagnosis codes, I48.0, I48.1x, I48.2x, I48.91, or two outpatient visits with a principal or secondary discharge diagnosis of AF during index period, (3) hospitals had continuous data submission during the look-back and follow-up periods, and (4) patients with claims linkage who were eligible for medical and pharmacy benefits during the 6-month look-back period, index visit and follow-up period. Patients were excluded if (1) they had ≥1 visit(s) with a principal or secondary discharge diagnosis of AF during the look-back period, (2) patients with evidence of OAC use during the look-back period, (3) patients with evidence of hip/knee replacement surgery within six weeks before the index date (the admission date of the index visit) due to the potential changes in treatment to prevent venous thromboembolism post-surgery, (4) patients with evidence of valvular heart disease (ICD-10-CM codes: mitral stenosis I05.x, I08.0, I08.8, I08.9, I34.x and tetralogy of Fallot Q21.3), heart valve replacement/transplant, venous thromboembolism, cardiac surgery, pericarditis, hyperthyroidism, thyrotoxicity, or pregnancy anytime during the look-back period or the index visit.

### 2.3 Study measures

Baseline characteristics assessed at the index visit included demographics (age, sex, race/ethnicity), primary payer, visit characteristics (care setting, admission point of origin, admission type, discharge status), and hospital characteristics (geographic region, urbanicity, teaching status, and number of beds). CHA_2_DS_2_-VASc Score for Atrial Fibrillation Stroke Risk was calculated based on age, sex, congestive heart failure, hypertension, stroke/transient ischemic attack/thromboembolism, diabetes, and vascular disease history (23). These conditions were assessed during the look-back period and index visit. HAS-BLED Score was calculated based on hypertension, abnormal renal/liver function, stroke, bleeding history or predisposition, elderly, drugs and alcohol. These conditions were assessed during the look-back period and index visit. Similar to other real-world studies (24, 25), the calculation did not include the labile international normalized ratio because of the unavailability of this information in the database. Comorbidities and the Deyo-modified Charlson Comorbidity Index (CCI) (26, 27) were assessed using patient-level data from the index visit and the look-back period (Supplementary Table S2).

Furthermore, the timing of OAC initiation was evaluated. It was categorized based on whether the OAC was initiated at index visit (i.e., filling a prescription for an OAC during their index visit or within a 7-day grace period post-index discharge) or during follow-up (i.e., filling a prescription for an OAC after this 7-day period but within one year of index discharge). The study further assessed the initiation of OAC within specific time frames post-index discharge, specifically within 30, 60, and 90 days for patients who did not initiate OAC at index visit as well as the type of OAC used. All-cause and stroke/SE-specific hospitalizations (i.e., those where the primary diagnosis was stroke or SE) during the follow-up period were assessed. Additionally, the primary diagnosis for all-cause hospitalizations during the follow-up period was evaluated.

### 2.4 Statistical analysis

Descriptive statistics described demographics and OAC utilization. The association between OAC initiation timing and the one-year risk of hospitalizations was analyzed using descriptive and multivariable adjusted analyses. These analyses compared the hospitalization risks between two cohorts: (1) OAC initiated at index visit, and (2) OAC initiated during follow-up. Multivariable logistic regression adjusted for potential confounders, including patient demographics (age, sex, race, ethnicity), the primary payer, care setting of index visit, hospital characteristics (urban/rural, geographic region, hospital size, teaching status), CCI score, obesity, diabetes, vascular disease history, and the CHA_2_DS_2_-VASc score. Sensitivity analyses were conducted by including additional variables, specifically renal disease, dementia, any malignancy, admission type, and discharge status. An additional sensitivity analysis was conducted on patients using DOACs, excluding those treated with warfarin, and by stratifying the results by prior stroke history. Another sensitivity analysis was conducted on patients with at least one refill during follow-up. Collinearity was assessed using variance inflation factor (VIF), where a VIF greater than 5 was considered as collinearity. Continuous variables were presented as means and standard deviations, and compared using *t-tests* or Wilcoxon Rank Sum test for non-normal distributions. Categorical variables were expressed as counts and percentages, and compared using *Chi-square* tests. A *p-value* < 0.05 was considered significant for determining differences between cohorts. Analyses were performed using R 3.6.3 (28).

## 3 Results

Between January 1, 2017, and March 31, 2021, we identified 93,251,105 patients aged ≥18. Of these, 23,148 adults with newly diagnosed NVAF were included (Supplementary Table S3). 11,059 (47.8%) received OAC within one year of diagnosis (OAC users), while 12,089 (52.2%) did not (Non-OAC users). The lowest OAC initiation rate (44.5%) was in 2017, and the highest (52.3%) in 2021.

The mean age was 70 ± 14 years (Table 1, median = 70; 62.7% ≥ 65 years), with 46.9% females, and 80.0% white. The major healthcare coverage was Medicare (63.4%), followed by commercial (21.0%), Medicaid (13.2%), and other, uninsured, or unknown (2.4%). The median CCI was 3. Common comorbidities included coronary artery disease (44.2%) and congestive heart failure (39.7%). The median CHA_2_DS_2_-VASc score was 4, with 77.8% of males and 85.4% of females having CHA_2_DS_2_-VASc scores of ≥2 and ≥3, respectively. The median HAS-BLED score was 3, with 60.4% patients having HAS-BLED score ≥3, and 74.1% of them diagnosed at an inpatient setting. Most patients were admitted from non-healthcare facilities (77.4%), came in for an emergency visit (63.8%), and were discharged to home (64.1%). The characteristics of hospitals, where patients received their NVAF diagnosis, varied (Table 2). Most patients were treated in urban (86.7%), non-teaching hospitals (54.7%), with bed size of 1–299 (38.8%).

**Table 1:** Baseline demographic, clinical and visit characteristics overall and by OAC use.

Baseline characteristics	Overall	OAC users	Non-OAC users	*p-value*
	(*n* = 23,148)	(*n* = 11,059)	(*n* = 12,089)	
Age, years Mean (SD)	70 (14)	70 (13)	69.5 (15)	0.6871
Age group, *n* (%)				<0.0001
18–54	3,149 (13.6%)	1,305 (11.8%)	1,844 (15.3%)	
35–64	5,479 (23.7%)	2,741 (24.8%)	2,738 (22.6%)	
65–74	5,331 (23.0%)	2,733 (24.7%)	2,598 (21.5%)	
75+	9,189 (39.7%)	4,280 (38.7%)	4,909 (40.6%)	
Female, *n* (%)	10,865 (46.9%)	5,202 (47.0%)	5,663 (46.8%)	0.7672
Race, *n* (%)				0.0366
White	18,522 (80.0%)	8,789 (79.5%)	9,733 (80.5%)	
Black	2,398 (10.4%)	1,159 (10.5%)	1,239 (10.2%)	
Asian	426 (1.8%)	195 (1.8%)	231 (1.9%)	
Other/unknown	1,802 (7.8%)	916 (8.3%)	886 (7.3%)	
Ethnicity, *n* (%)				<0.0001
Hispanic or Latino	1,767 (7.6%)	868 (7.8%)	899 (7.4%)	
Not Hispanic or Latino	17,368 (75.0%)	8,436 (76.3%)	8,932 (73.9%)	
Other/Unknown	4,013 (17.3%)	1,755 (15.9%)	2,258 (18.7%)	
Primary payer, *n* (%)				<0.0001
Medicare	14,678 (63.4%)	7,031 (63.6%)	7,647 (63.3%)	
Medicaid	3,059 (13.2%)	1,351 (12.2%)	1,708 (14.1%)	
Commercial	4,859 (21.0%)	2,439 (22.1%)	2,420 (20.0%)	
Uninsured	163 (0.7%)	77 (0.7%)	86 (0.7%)	
Unknown	389 (1.7%)	161 (1.5%)	228 (1.9%)	
CCI				<0.0001
Mean (SD)	3.53 (3)	3.58 (3)	3.49 (3)	
Median (IQR)	3 (1, 5)	3 (1, 5)	3 (1, 5)	
Major comorbidities, *n* (%)^a^				
Coronary artery disease	10,230 (44.2%)	5,110 (46.2%)	5,120 (42.4%)	<0.0001
Congestive heart failure	9,195 (39.7%)	4,979 (45.0%)	4,216 (34.9%)	<0.0001
Chronic pulmonary disease	9,067 (39.2%)	4,406 (39.8%)	4,661 (38.6%)	0.0454
Diabetes	8,807 (38.0%)	4,516 (40.8%)	4,291 (35.5%)	<0.0001
Obesity	8,241 (35.6%)	4,447 (40.2%)	3,794 (31.4%)	<0.0001
Vascular disease history	8,131 (35.1%)	3,998 (36.2%)	4,133 (34.2%)	0.0018
Renal disease	6,779 (29.3%)	3,325 (30.1%)	3,454 (28.6%)	0.0126
Cerebrovascular disease	5,459 (23.6%)	2,758 (24.9%)	2,701 (22.3%)	<0.0001
Myocardial infarction	4,675 (20.2%)	2,323 (21.0%)	2,352 (19.5%)	0.0033
Peripheral vascular disease	3,951 (17.1%)	1,878 (17.0%)	2,073 (17.1%)	0.7371
Any malignancy	3,002 (13.0%)	1,270 (11.5%)	1,732 (14.3%)	<0.0001
Dementia	2,957 (12.8%)	1,183 (10.7%)	1,774 (14.7%)	<0.0001
Stroke/SE	2,554 (11.0%)	1,439 (13.0%)	1,115 (9.2%)	<0.0001
CHA_2_DS_2_-VASc Score				<0.0001
Mean (SD)	3.72 (2)	3.87 (2)	3.58 (2)	
Median (IQR)	4 (2, 5)	4 (3, 5)	4 (2, 5)	
CHA_2_DS_2_-VASc Score—Male				<0.0001
0	914 (7.4%)	330 (5.6%)	584 (9.1%)	
1	1,818 (14.8%)	692 (11.8%)	1,126 (17.5%)	
≥2	9,551 (77.8%)	4,835 (82.6%)	4,716 (73.4%)	
CHA_2_DS_2_-VASc Score—Female				<0.0001
1	514 (4.7%)	164 (3.2%)	350 (6.2%)	
2	1,069 (9.8%)	465 (8.9%)	604 (10.7%)	
≥3	9,282 (85.4%)	4,573 (87.9%)	4,709 (83.2%)	
HAS-BLED score				0.8016
Mean (SD)	2.91 (1)	2.91 (1)	2.91 (1)	
Median (IQR)	3 (2, 4)	3 (2, 4)	3 (2, 4)	
HAS-BLED Score				<0.0001
0	969 (4.2%)	369 (3.3%)	600 (5.0%)	
1	2,939 (12.7%)	1,348 (12.2%)	1,591 (13.2%)	
2	5,259 (22.7%)	2,627 (23.8%)	2,632 (21.8%)	
≥3	13,981 (60.4%)	6,715 (60.7%)	7,266 (60.1%)	
Inpatient diagnosis visit, *n* (%)	17,164 (74.1%)	8,776 (79.4%)	8,388 (69.4%)	<0.0001
Admission point of origin, *n* (%)				<0.0001
Non-healthcare facility (e.g., home)	17,926 (77.4%)	8,799 (79.6%)	9,127 (75.5%)	
Clinic	3,053 (13.2%)	1,238 (11.2%)	1,815 (15.0%)	
Transferred from acute care facility	1,635 (7.1%)	841 (7.6%)	794 (6.6%)	
Transferred from intermediate care or skilled nursing facility	212 (0.9%)	82 (0.7%)	130 (1.1%)	
Other/unknown	322 (1.4%)	99 (0.9%)	223 (1.8%)	
Admission type, *n* (%)				<0.0001
Elective	5,304 (22.9%)	2,037 (18.4%)	3,267 (27.0%)	
Urgent	2,196 (9.5%)	1,145 (10.4%)	1,051 (8.7%)	
Emergency	14,766 (63.8%)	7,541 (68.2%)	7,225 (59.8%)	
Trauma or injury center	167 (0.7%)	62 (0.6%)	105 (0.9%)	
Other unknown	715 (3.1%)	274 (2.5%)	441 (3.6%)	
Discharge status, *n* (%)				<0.0001
Home	14,842 (64.1%)	7,133 (64.5%)	7,709 (63.8%)	
Home health	2,931 (12.7%)	1,569 (14.2%)	1,362 (11.3%)	
Transferred to intermediate care, skilled nursing or other long-term care facility	3,947 (17.1%)	1,875 (17.0%)	2,072 (17.1%)	
Transferred to acute care facility	60 (0.3%)	36 (0.3%)	24 (0.2%)	
Hospice	246 (1.1%)	65 (0.6%)	181 (1.5%)	
Expired	129 (0.6%)	18 (0.2%)	111 (0.9%)	
Other/unknown	993 (4.3%)	363 (3.3%)	630 (5.2%)	

OAC, oral anticoagulants; SD, standardized deviation; IQR, interquartile range; CCI, Charlson comorbidities.

^a^ Includes Charlson comorbidities with a prevalence >5%, as well as cardiovascular diseases and related risk factors such as obesity, stroke/SE, history of vascular disease, and coronary artery disease.

**Table 2:** Hospital characteristics at initial NVAF diagnosis, overall and by OAC use.

Hospital characteristics	Overall	OAC users	Non-OAC users	*p-value*
	(*n* = 23,148)	(*n* = 11,059)	(*n* = 12,089)	
Hospital Setting, *n* (%)				<0.0001
Urban	20,074 (86.7%)	9,733 (88.0%)	10,341 (85.5%)	
Rural	3,074 (13.3%)	1,326 (12.0%)	1,748 (14.5%)	
Teaching Status, *n* (%)				0.0019
Teaching	10,494 (45.3%)	5,131 (46.4%)	5,363 (44.4%)	
Non-teaching	12,654 (54.7%)	5,928 (53.6%)	6,726 (55.6%)	
Geographic Region, *n* (%)				0.0036
Northeast	3,008 (13.0%)	1,482 (13.4%)	1,526 (12.6%)	
Midwest	5,210 (22.5%)	2,445 (22.1%)	2,765 (22.9%)	
South	10,841 (46.8%)	5,262 (47.6%)	5,579 (46.1%)	
West	4,089 (17.7%)	1,870 (16.9%)	2,219 (18.4%)	
Bed size, *n* (%)				<0.0001
1–299	8,965 (38.8%)	4,084 (37.0%)	4,881 (40.4%)	
300–499	7,116 (30.8%)	3,458 (31.3%)	3,658 (30.3%)	
500+	7,030 (30.4%)	3,501 (31.7%)	3,529 (29.2%)	

NVAF, nonvalvular atrial fibrillation; OAC, oral anticoagulants.

When stratified by OAC users and non-OAC users, age, sex, and race distributions were similar between the two groups. Compared to non-OAC users, a lower proportion of OAC users were Hispanic or Latino and on Medicaid, while a higher percentage had commercial payers (*p-value* < 0.0001). In terms of comorbidities, OAC users generally had a higher prevalence of cardiovascular disease and risk factors than non-OAC users, such as coronary artery disease (46.2% vs. 42.4%), congestive heart failure (45.0% vs. 34.9%), diabetes (40.8% vs. 35.5%), obesity (40.2% vs. 31.4%), vascular disease history (36.2% vs. 34.2%), cerebrovascular disease (24.9% vs. 22.3%), myocardial infarction (21.0% vs. 19.5%), and stroke/SE (13.0% vs. 9.2%). In contrast, non-OAC users had a higher prevalence of malignancy (14.3% vs. 11.5%) and dementia (14.7% vs. 10.7%) than OAC users. The CHA_2_DS_2_-VASc Score was higher among OAC users, while the HAS-BLED score was 3 in both groups. A larger proportion of OAC users vs. non-OAC users (were diagnosed with AF in the inpatient setting (79.4% vs. 69.4, respectively, *p-value* < 0.0001), with higher emergency admissions of (68.2% vs. 59.8%, respectively), and higher elective admissions (18.4% vs. 27.0%, respectively, *p-value* < 0.0001). Compared to non-OAC users, more OAC users were discharged to home (*p-value* < 0.0001). Non-OAC users were more frequently discharged to hospice or were documented as expired than OAC users (*p-value* < 0.0001).

Among OAC users, 74.9% initiated OAC during the index visit, with the remaining 25.1% initiating during the one-year follow-up (Table 3). When stratified by diagnosis visit care setting, 84.3% of patients in an inpatient setting initiated OAC during the index visit, compared to 39.0% of patients in an outpatient setting. Among patients initiating OAC during follow-up, the median time to OAC initiation following diagnosis was 94 days, with no significant differences between those diagnosed in inpatient and those in outpatient settings. Apixaban was the most commonly used OAC (62.2%), followed by rivaroxaban (23.2%), warfarin (15.8%), and dabigatran (3.1%). Throughout this follow-up period, a total of 3,695 (33.4%) OAC users experienced all-cause hospitalization, of which 177 were due to stroke/SE (Table 4). Among those initiating OAC at the index visit, the 12-month risk was 29.2% for all-cause hospitalization and 1.3% for hospitalization due to stroke/SE. In contrast, among those initiating OAC during follow-up, 1,274 (45.9%) and 73 (2.6%) experienced all-cause and stroke/SE hospitalization, respectively. The adjusted odds ratio for all-cause hospitalization was 0.35 (95% CI: 0.32–0.39, *p-value* < 0.0001) and, for hospitalization due to stroke/SE, it was 0.34 (95% CI: 0.24–0.47, *p-value* < 0.0001), comparing those initiating OAC at the index visit to those during the follow-up period. When stratified by diagnostic visit care setting, both all-cause hospitalization and hospitalization due to stroke/SE remained lower for those initiating at the index visit compared to during the follow-up period (Inpatient: adjusted OR (95% CI): all-cause hospitalization 0.31 (0.27–0.35); hospitalization due to stroke/SE 0.36 (0.25–0.52); Outpatient: adjusted OR (95% CI): all-cause hospitalization 0.51 (0.42–0.63); hospitalization due to stroke/SE 0.18 (0.05–0.61)). The observed trend of a lower hospitalization rate in those initiating OAC at the index visit compared to during follow-up remained after excluding warfarin users (Supplementary Table S4), stratifying by stroke history (Supplementary Table S5), including additional variables in the model (Supplementary Table S6), and among patients with at least one refill (Supplementary Table S7). Among OAC users, the primary diagnoses leading to all-cause hospitalizations during the follow-up period included atrial fibrillation (20.0%), sepsis (12.3%), hypertensive heart and chronic kidney disease (9.4%), hypertensive heart disease (7.3%), and acute kidney failure (4.5%), as detailed in Table 5.

**Table 3:** OAC initiation, timing and type among OAC users, overall and by diagnosis visit care setting.

OAC use	With OAC use	Inpatient	Outpatient	*p-value*
	(*n* = 11,059)	(*n* = 8,776)	(*n* = 2,283)	
OAC initiation during				<0.0001
Index visit, *n* (%)	8,285 (74.9%)	7,394 (84.3%)	891 (39.0%)	
Follow-up period, *n* (%)	2,774 (25.1%)	1,382 (15.7%)	1,392 (61.0%)	
Within *x* days of the index discharge date, *n* (%)				
30 days	639 (5.8%)	319 (3.6%)	320 (14.0%)	<0.0001
60 days	1,093 (9.9%)	555 (6.3%)	538 (23.6%)	<0.0001
90 days	1,358 (12.3%)	681 (7.8%)	677 (29.7%)	<0.0001
Time to OAC initiation from index discharge date, days				0.979
Mean (SD)	127.3 (106.7)	127.5 (107.8)	127.1 (105.6)	
Median (IQR)	94 (33, 210.5)	94 (33, 206)	94 (32, 211)	
Type of OAC, *n*(%)				
Warfarin	1,752 (15.8%)	1,421 (16.2%)	331 (14.5%)	0.0484
Apixaban	6,878 (62.2%)	5,519 (62.9%)	1,359 (59.5%)	0.0032
Rivaroxaban	2,568 (23.2%)	1,906 (21.7%)	662 (29.0%)	<0.0001
Dabigatran	344 (3.1%)	275 (3.1%)	69 (3.0%)	0.7851
Edoxaban	2 (0.02%)	2 (0.02%)	0 (0.0%)	0.4707

OAC, oral anticoagulants; SD, standardized deviation; IQR, interquartile range.

**Table 4:** Time to initiate OAC and hospitalization during follow-up among OAC users.

Hospitalizations	With OAC use	OAC initiated at index visit	OAC initiated during follow-up	*p*-value
**Overall**	**11,059**	**8,285**	**2,774**	
*All-cause hospitalization*	3,695 (33.4%)	2,421 (29.2%)	1,274 (45.9%)	
* *Unadjusted OR	0.49 (0.44–0.53)			<0.001
* *Adjusted OR^a^	0.35 (0.32–0.39)			<0.001
*Hospitalization due to stroke/SE*	177 (1.6%)	104 (1.3%)	73 (2.6%)	
Unadjusted OR	0.47 (0.35–0.64)			<0.001
Adjusted OR^a^	0.34 (0.24–0.47)			<0.001
**Inpatient diagnostic visit**	**8,776**	**7,394**	**1,382**	
*All-cause hospitalization*	2,985 (34.0%)	2,216 (30.0%)	769 (55.6%)	
Unadjusted OR	0.34 (0.30–0.38)			<0.001
Adjusted OR^b^	0.31 (0.27–0.35)			<0.001
*Hospitalization due to stroke/SE*	148 (1.3%)	101 (1.4%)	47 (3.4%)	
Unadjusted OR	0.39 (0.28–0.56)			<0.001
Adjusted OR^b^	0.36 (0.25–0.52)			<0.001
**Outpatient diagnostic visit**	**2,283**	**891**	**1,392**	
*All-cause hospitalization*	710 (31.1%)	205 (23.0%)	505 (36.3%)	
Unadjusted OR	0.52 (0.43–0.64)			<0.001
Adjusted OR^b^	0.51 (0.42–0.63)			<0.001
*Hospitalization due to stroke/SE*	29 (1.3%)	3 (0.0%)	26 (1.9%)	
Unadjusted OR	0.18 (0.05–0.59)			0.005
Adjusted OR^b^	0.18 (0.05–0.61)			0.006

OAC, oral anticoagulants; OR, odds ratio.

Bold values denote the number of patients on OAC, both overall and stratified by initiation time.

^a^ Adjusted for Age Category, Gender, Race, Ethnicity, Primary Payor, Care setting of index visit, Hospital Characteristics (Urban/rural, Geographic Region, Hospital Size, Teaching Status), Charlson Comorbidity Index, Obesity, Diabetes, Vascular disease history, and CHA_2_DS_2_-VASc score.

^b^ Adjusted for Age Category, Gender, Race, Ethnicity, Primary Payor, Hospital Characteristics (Urban/rural, Geographic Region, Hospital Size, Teaching Status), Charlson Comorbidity Index, Obesity, Diabetes, Vascular disease history, and CHA_2_DS_2_-VASc score.

**Table 5:** Top primary diagnosis of all-cause hospitalization during follow-up period among OAC users.

Diagnosis	Total number
All-cause hospitalization	3,695
Atrial fibrillation	739 (20.0%)
Sepsis	454 (12.3%)
Hypertensive heart and chronic kidney disease	347 (9.4%)
Hypertensive heart disease	268 (7.3%)
Acute kidney failure	167 (4.5%)
Other chronic obstructive pulmonary disease	142 (3.8%)
Respiratory failure, not elsewhere classified	135 (3.7%)
Pneumonia	126 (3.4%)
Stroke	124 (3.4%)
Acute myocardial infarction	90 (2.4%)
Urinary tract infection	75 (2.0%)

## 4 Discussion

Using real-world data, this study found that, among patients newly diagnosed with NVAF, 47.8% initiated OAC initiation within one year. It appears that patients with malignancy and dementia were associated with lower odds of initiating OAC within one year post diagnosis. Additionally, for patients who initiated OAC within one year post AF diagnosis, an early initiation was associated with lower risk of all-cause and stroke/SE-specific hospitalization.

The recommendation for using OAC in AF patients has been well-established for years. Both the 2019 American Heart Association/American College of Cardiology/Heart Rhythm Society (4) and the 2020 European Society of Cardiology guidelines (29) have similar recommendations for managing AF, emphasizing the use of the CHA_2_DS_2_-VASc score for stroke risk stratification. For patients with higher scores (≥2 for men, ≥3 for women), there is a consensus recommendation to initiate oral anticoagulant (OAC) therapy to mitigate the stroke risk, while for those at intermediate risk (1 for men, 2 for women), OAC prescription remains a consideration. For low-risk patients (0 for men, 1 for women), it is reasonable to omit anticoagulant therapy, given their relatively low risk of stroke. Additionally, both guidelines highlight the importance of balancing potential stroke benefits against the elevated bleeding risk associated with OAC use. Despite these guidelines, OAC use in the AF population remains suboptimal. One study demonstrated a modest increasing trend in OAC initiation from 2010 through 2020 in the Medicare Advantage population, with a peak of 32.9% in 2020 for newly diagnosed AF patients with elevated stroke risk (CHA_2_DS_2_-VASc Score ≥2 for men, CHA_2_DS_2_-VASc Score ≥3 for women) (11). Another study within the fee-for-service Medicare population between 2013 and 2017 showed a 48.7% OAC utilization rate in patients with a CHA_2_DS_2_-VASc Score of ≥2 (12). Furthermore, a study from 2011–2020 in adults aged over 40 with AF across 88 US health systems reported a slight increase in OAC usage over the years, from 56.3%–64.7%. (13) Our study found only 47.8% OAC-naïve NVAF patients initiated OAC within one year following the AF diagnosis, with the rate of OAC initiation among patients with elevated stroke risk (CHA_2_DS_2_-VASc Score ≥2 for men, CHA_2_DS_2_-VASc Score ≥3 for women) at 50.0%. These findings suggest variation in OAC usage rates across different populations. In addition, despite the general upward trend observed in recent years, these findings highlight the need for enhanced strategies to improve adherence to guideline-recommended OAC use in AF patient, aiming for optimal stroke risk management. Notably, significant disparities in OAC prescription rates have been observed among high-risk AF patients managed by primary care clinicians within the same regional health system (30). Implementing targeted interventions, such as an email-based educational initiative directed at primary care clinicians managing high-risk AF patients, has been shown to effectively increase OAC prescription rates and improve overall stroke prevention in this population (30).

In addition, some patients with low CHA_2_DS_2_-VASc scores were also found to be taking OAC (36.1% for men with a CHA_2_DS_2_-VASc score of 0, and 31.9% for women with a CHA_2_DS_2_-VASc score of 1). It is possible that CHA_2_DS_2_-VASc scores are underreported in this study. Our look-back period was 365 days, which may have led to missing vascular events that occurred earlier. Additionally, CHA_2_DS_2_-VASc scores were assessed at baseline and could have increased during the follow-up period, during which OAC might have been initiated. Despite these factors, the current finding aligns with previous studies that also reported higher-than-expected OAC use in populations with low CHA_2_DS_2_-VASc scores. A study in Australia reported that 25.1% of AF patients with low stroke risk (CHA_2_DS_2_-VASc score of 0 for males and 1 for females) had a record of OAC prescription within 60 days of AF diagnosis (31). Another study in the US using the PINNACLE registry found OAC use in 31.1% of AF patients with a CHA_2_DS_2_-VASc score of 0 and 34.6% with a CHA_2_DS_2_-VASc score of 1 (32). Further research is necessary to better understand benefits and potential risks of OAC use in patients with low CHA_2_DS_2_-VASc scores.

Studies have investigated demographic and clinical characteristics influencing the OAC use, reporting that factors such as older age, female, Black race, and certain comorbidities including dementia, frailty, anemia, bleeding were associated with decreased odds of OAC initiation (11, 12). In contrast, histories of cardiovascular diseases, including stroke, SE, and obesity, were associated with increased likelihood of OAC prescription. Interestingly, there was no substantial difference in CHA_2_DS_2_-VASc score and HAS-BLED scores between OAC users and non-users. The current study aligns with these observations. Despite similar demographic characteristics between OAC users and non-users, a distinct comorbidity profile was observed: OAC users showed a higher prevalence of cardiovascular disease and risk factors, whereas non-OAC users had a higher prevalence of malignancy and dementia. The CHA_2_DS_2_-VASc score was slightly higher among OAC users, with no notable difference in HAS-BLED scores between the groups. These insights underline the complexity of decision-making in anticoagulant therapy and highlight the necessity for a nuanced approach that considers individual patient profiles beyond risk scores alone. This could be pivotal in improving OAC utilization where it is most needed, potentially optimizing therapeutic outcomes in atrial fibrillation management.

The current study emphasized that initiating OAC early might be associated with a decreased risk of all-cause hospitalization and hospitalization due to stroke/SE. Although few studies have explored the association between the timing of OAC initiation and hospitalization risk, this observation is in line with findings from a 3-year follow-up study, which demonstrated that OAC use was associated with a 7% lower risk of all-cause hospitalization compared to non-use among Medicare beneficiaries with AF (33). Although studies specifically examining NVAF patients without prior stroke are limited, there is substantial literature assessing OAC timing for those with NVAF and concurrent stroke (14–17). These studies largely indicated similar safety and effectiveness regardless of whether OAC therapy initiates earlier or later. A meta-analysis comprising six cohort studies and two randomized controlled trials reported no significant difference in the safety and efficacy of OACs initiated within one to two weeks following an acute ischemic stroke (16). Consistent with these findings, the Early vs. Late Initiation of Direct Oral Anticoagulants in Post-ischemic Stroke Patients with Atrial Fibrillation (ELAN) trial showed no significant difference in the combined outcomes, including recurrent ischemic stroke, systemic embolism, major bleeding, symptomatic intracranial hemorrhage, or vascular death, within 30 days of starting early vs. later direct oral anticoagulant (DOAC) administration in patients with NVAF and acute ischemic stroke (OR 0.70, 95% CI 0.44–1.14) (14). Interestingly, a lower risk was observed at the 90-day interval (OR 0.65, 95% CI 0.42–0.99) (14). Other studies, including the TIMING study, a registry-based randomized controlled noninferiority trial, have similarly found no substantial association between the timing of OAC initiation and stroke outcomes following an acute ischemic stroke (15, 17). In the current study, stratified by stroke history, NVAF patients would benefit from initiating OAC earlier regarding stroke/SE with or without stroke history. These collective insights suggest that while the early initiation of OACs might not necessarily amplify adverse outcomes, its advantages, especially in patients without prior stroke, are yet to be conclusively determined. This highlights an evident gap in knowledge and the pressing need for further investigations into the timing of OAC initiation in NVAF patients without a stroke history.

It is noteworthy that the majority of hospitalizations during the follow-up period were not due to stroke or SE. The most prevalent primary diagnosis remains to be atrial fibrillation, followed by sepsis, heart and kidney diseases, and pulmonary diseases. This pattern suggested that patients with NVAF may have a heightened risk of comorbidities and other underlying factors contributing to their overall likelihood of hospitalization. These observations highlight the complexity of the NVAF population and emphasize the urgent need for comprehensive management strategies to effectively mitigate their stroke risk.

This study is not without limitations. Due to its retrospective observational design, it can identify associations but cannot establish causality. There may be residual confounding even after adjustment, such as the subjective nature of anticoagulant selection by physicians, who decide both when and what specific type of OAC to use. Additionally, there is a possibility that patients who started OAC earlier were more health-conscious, which could skew results. Additionally, the reliance on administrative data introduces potential constraints, such as the possibility of coding errors and data incompleteness. For example, HAS-BLED score has labile INR, which is not available in the data, and while NVAF has different forms, their characteristics (e.g., diagnostic modalities and whether it is clinical or subclinical, or with or without symptoms) are not available in this data. Our study utilizes hospital administrative data and closed claims data from a real-world database, which serve as proxies for OAC utilization but may not accurately reflect actual usage or the exact date when the patient started taking the OAC. However, sensitivity analysis among patients with at least one refill of OAC showed similar results, suggesting that the findings are robust among patients likely to be actually taking the OAC. Moreover, this study does not assess OAC discontinuation patterns, such as cessation after successful AF ablation, which may influence outcomes. Despite these limitations, this study, using real-world data, described the recent trends in OAC utilization among newly diagnosed NVAF patients. Moreover, it is the first study to investigate the association between the timing of OAC initiation and the rates of all-cause and stroke/SE-specific hospitalizations, using real-world data. Future studies are needed to further investigate drug adherence, different types of NVAF and their characteristics, and OAC dosing to better assess their impact on the association between OAC and hospitalization in the NVAF population. Future studies should further explore drug adherence, NVAF subtypes and characteristics, and OAC dosing to better understand their impact on hospitalization risks. Additionally, research should evaluate whether healthcare interventions, such as closer follow-up and increased awareness, modify this association. Lastly, assessing follow-up period characteristics, such as fall risk and hemorrhagic events, could provide further insights into patient outcomes across treatment groups.

In conclusion, this study demonstrated the underutilization of OAC among patients with NVAF, despite existing guideline recommendations. Furthermore, our findings suggest that early initiation of OAC therapy could be associated with decreased rates of hospitalization. The findings underscored a compelling need for personalized, comprehensive management strategies for NVAF patients, taking into account not only stroke risk but also their comorbidity profile. The observed correlation between the timing of OAC initiation and reduced hospitalization rates warrants further investigation, especially in patients without stroke history.

## Data Availability

The data analyzed in this study was obtained from the Premier Healthcare Database and its linked claims database. The following licenses/restrictions apply: they are proprietary databases that can be accessed by purchasing an applicable license. Requests to access these datasets should be directed to Dr. Chendi Cui (chendi_cui@premierinc.com).
