# Supplementary material for: Oral anticoagulant timing and hospitalization in newly diagnosed nonvalvular atrial fibrillation patients
Source: Front Cardiovasc Med. 2025 Apr 4;12:1522154. doi: 10.3389/fcvm.2025.1522154 (PMC12006160; doi:10.3389/fcvm.2025.1522154)
Supplement: Supplementary file 1 [file Datasheet1.pdf]

Supplementary Material

Supplementary Methods, Figure and Tables

This supplementary material has been provided by the authors to give readers additional information about their work.

Supplementary Methods

Supplementary Figures and Tables

Supplementary Figure. Study timeline

Supplementary Table 1.

Supplementary Table 2. Charlson comorbidity index diagnosis and procedure codes

Supplementary Table 3. Patient attrition

Supplementary Table 4. Time to initiate OAC and hospitalization during follow-up among OAC users, excluding Warfarin users

Supplementary Table 5. Time to initiate OAC and hospitalization during follow-up among OAC users, by stroke history

Supplementary Methods

Data Source

The Premier Healthcare Database (PHD) includes inpatient and hospital-based outpatient discharge information from more than 1200 US hospitals and 20-25% of all US inpatient admissions from geographically diverse, non-governmental community and teaching hospitals.[1] The PHD includes data on patient demographics and disease states, health insurance type, admission and discharge diagnoses, admission source and type, discharge status, and disposition. Unique masked identifiers track patients in the same hospital across inpatient and hospital-based outpatient settings. For most PHD data elements, less than one percent of patient records having missing information and for key elements, such as demographics and diagnostic information, less than 0.01 percent have missing data.

Supplementary Figure. Study timeline

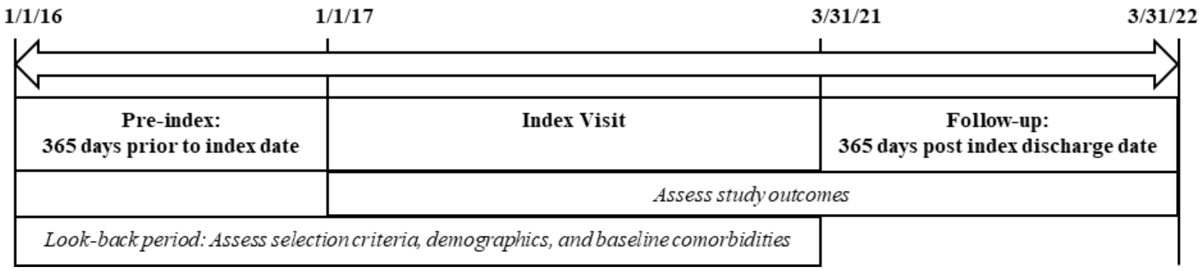

Supplementary Table 1. STROBE Statement—Checklist of items that should be included in reports of cohort studies

	Item No	Recommendation	Page No
**Title and abstract**	1	(*a*) Indicate the study's design with a commonly used term in the title or the abstract<br>(*b*) Provide in the abstract an informative and balanced summary of what was done and what was found	Title and abstract
**Introduction**			
Background/rationale	2	Explain the scientific background and rationale for the investigation being reported	Introduction
Objectives	3	State specific objectives, including any prespecified hypotheses	Introduction
**Methods**			
Study design	4	Present key elements of study design early in the paper	Methods
Setting	5	Describe the setting, locations, and relevant dates, including periods of recruitment, exposure, follow-up, and data collection	Methods
Participants	6	(*a*) Give the eligibility criteria, and the sources and methods of selection of participants. Describe methods of follow-up<br>(*b*) For matched studies, give matching criteria and number of exposed and unexposed	Methods<br>Methods
Variables	7	Clearly define all outcomes, exposures, predictors, potential confounders, and effect modifiers. Give diagnostic criteria, if applicable	Methods
Data sources/ measurement	8*	For each variable of interest, give sources of data and details of methods of assessment (measurement). Describe comparability of assessment methods if there is more than one group	Methods
Bias	9	Describe any efforts to address potential sources of bias	Methods
Study size	10	Explain how the study size was arrived at	Methods
Quantitative variables	11	Explain how quantitative variables were handled in the analyses. If applicable, describe which groupings were chosen and why	Methods
Statistical methods	12	(*a*) Describe all statistical methods, including those used to control for confounding<br>(*b*) Describe any methods used to examine subgroups and interactions<br>(*c*) Explain how missing data were addressed<br>(*d*) If applicable, explain how loss to follow-up was addressed	Methods

		(*e*) Describe any sensitivity analyses	
**Results**			
Participants	13*	(a) Report numbers of individuals at each stage of study—eg numbers potentially eligible, examined for eligibility, confirmed eligible, included in the study, completing follow-up, and analysed	Results
		(b) Give reasons for non-participation at each stage	Results
		(c) Consider use of a flow diagram	Results
Descriptive data	14*	(a) Give characteristics of study participants (eg demographic, clinical, social) and information on exposures and potential confounders	Results
		(b) Indicate number of participants with missing data for each variable of interest	Results
		(c) Summarise follow-up time (eg, average and total amount)	Results
Outcome data	15*	Report numbers of outcome events or summary measures over time	Results
Main results	16	(*a*) Give unadjusted estimates and, if applicable, confounder-adjusted estimates and their precision (eg, 95% confidence interval). Make clear which confounders were adjusted for and why they were included	Results
		(*b*) Report category boundaries when continuous variables were categorized	Results
		(*c*) If relevant, consider translating estimates of relative risk into absolute risk for a meaningful time period	Results
Other analyses	17	Report other analyses done—eg analyses of subgroups and interactions, and sensitivity analyses	Results
**Discussion**			
Key results	18	Summarise key results with reference to study objectives	Discussion
Limitations	19	Discuss limitations of the study, taking into account sources of potential bias or imprecision. Discuss both direction and magnitude of any potential bias	Discussion
Interpretation	20	Give a cautious overall interpretation of results considering objectives, limitations, multiplicity of analyses, results from similar studies, and other relevant evidence	Discussion
Generalisability	21	Discuss the generalisability (external validity) of the study results	Discussion
**Other information**			
Funding	22	Give the source of funding and the role of the funders for the present study and, if applicable, for the original study on which the present article is based	Funding

*Give information separately for exposed and unexposed groups.

Note: An Explanation and Elaboration article discusses each checklist item and gives methodological background and published examples of transparent reporting. The STROBE checklist is best used in conjunction with this article (freely available on the Web sites of PLoS Medicine at http://www.plosmedicine.org/, Annals of Internal Medicine at http://www.annals.org/, and Epidemiology at http://www.epidem.com/). Information on the STROBE Initiative is available at http://www.strobe-statement.org.

Supplementary Table 2. Charlson comorbidity index diagnosis and procedure codes

Comorbidity	Type	ICD-10 codes
Myocardial infarction	Dx	I21.x, I22.x, I23.x, I25.2
Congestive heart failure	Dx	I50.x
Peripheral vascular disease	Dx	I73.9, I71.00, I71.01, I71.02, I71.03, I71.1, I71.2, I71.3, I71.4, I71.5, I71.6, I71.8, I71.9, I96, Z95.828
	Proc	04RK07Z, 04RK0JZ, 04RK0KZ, 04RK47Z, 04RK4JZ, 04RK4KZ, 04RL07Z, 04RL0JZ, 04RL0KZ, 04RL47Z, 04RL4JZ, 04RL4KZ, 04RM07Z, 04RM0JZ, 04RM0KZ, 04RM47Z, 04RM4JZ, 04RM4KZ, 04RN07Z, 04RN0JZ, 04RN0KZ, 04RN47Z, 04RN4JZ, 04RN4KZ, 04RP07Z, 04RP0JZ, 04RP0KZ, 04RP47Z, 04RP4JZ, 04RP4KZ, 04RQ07Z, 04RQ0JZ, 04RQ0KZ, 04RQ47Z, 04RQ4JZ, 04RQ4KZ, 04RR07Z, 04RR0JZ, 04RR0KZ, 04RR47Z, 04RR4JZ, 04RR4KZ, 04RS07Z, 04RS0JZ, 04RS0KZ, 04RS47Z, 04RS4JZ, 04RS4KZ, 04RT07Z, 04RT0JZ, 04RT0KZ, 04RT47Z, 04RT4JZ, 04RT4KZ, 04RU07Z, 04RU0JZ, 04RU0KZ, 04RU47Z, 04RU4JZ, 04RU4KZ, 04RV07Z, 04RV0JZ, 04RV0KZ, 04RV47Z, 04RV4JZ, 04RV4KZ, 04RW07Z, 04RW0JZ, 04RW0KZ, 04RW47Z, 04RW4JZ, 04RW4KZ, 04RY07Z, 04RY0JZ, 04RY0KZ, 04RY47Z, 04RY4JZ, 04RY4KZ
Cerebrovascular disease	Dx	I60.x, I61.x, I62.x, I63.x, I65.x, I66.x, I67.x, I68.x, I69.x, G45.x
Dementia	Dx	F03.90, F01.50, F01.51, F03.91, F02.80, F02.81
Chronic pulmonary disease	Dx	J40, J41.0, J41.1, J44.9, J44.0, J41.8, J42, J43.9, J45.20, J45.21, J45.22, J44.1, J45.990, J45.991, J45.909, J45.998, J45.902, J45.901, J47.9, J47.1, J67.0, J67.1, J67.2, J67.3, J67.4, J67.5, J67.6, J67.7, J67.8, J67.9, J60, J61, J62.8, J63.0, J63.1, J63.2, J63.3, J63.4, J63.5, J63.6, J66.0, J66.1, J66.2, J66.8, J64, J68.4
Rheumatic disease	Dx	M32.10, M34.0, M34.1, M34.9, M33.20, M06.9, M05.00, M05.30, M05.60, M06.1, M05.10, M35.3
Peptic ulcer disease	Dx	K25.0, K25.1, K25.2, K25.3, K25.4, K25.5, K25.6, K25.7, K25.9, K26.0, K26.1, K26.2, K26.3, K26.4, K26.5, K26.6, K26.7, K26.9, K27.0, K27.1, K27.2, K27.3, K27.4, K27.5, K27.6, K27.7, K27.9, K28.0, K28.1, K28.2, K28.3, K28.4, K28.5, K28.6, K28.7, K28.9
Mild liver disease	Dx	K70.30, K73.9, K73.0, K75.4, K73.2, K73.8, K74.0, K74.60, K74.69, K74.3, K74.4, K74.5
Diabetes without chronic complication	Dx	E11.9, E10.9, E13.9, E11.65, E10.65, E13.65, E10.1x, E11.1x, E13.1x, E11.0x, E13.0x, E11.64x, E10.64x, E13.64x
Diabetes with chronic complication	Dx	E10.2x, E10.3x, E10.4x, E10.5x, E10.61x, E10.62x, E10.63x, E10.69, E10.8, E11.2x, E11.3x, E11.4x, E11.5x, E11.61x,

		E11.62x, E11.63x, E11.69, E11.8, E13.2x, E13.3x, E13.4x, E13.5x, E13.61x, E13.62x, E13.63x, E13.69, E13.8
Hemiplegia or paraplegia	Dx	G04.1, G11.4, G80.1, G80.2, G81.x, G82.x, G83.0, G83.1x, G83.2x, G83.3x, G83.4, G83.9
Moderate or severe renal disease	Dx	I12.0, I13.11, I13.2, N03.2, N03.3, N03.5, N03.8, N03.9, N05.2 N05.5, N05.9, N08, N18.x, N19.x, N25.x, Z49.0x, Z49.3x, Z94.0, Z99.2
Any malignancy, including lymphoma and leukemia, except malignant neoplasm of skin	Dx	C00.x-C75.x (except C43.x and C44.x), C81.x-C85.x, C88.x, C90.x, C91.x-C95.x, C96.x, C7A.xxx, C7B.xxx, D00.xx, D01.xx, D02.x, D03.xx, D05.xx, D06.x, D07.xx, D09.xx, D47.9
Moderate or severe liver disease	Dx	I85.00, I85.01, I85.10, I85.11, I86.4, K70.4x, K71.1x, K71.7, K72.xx, K76.6, K76.7
Metastatic solid tumor	Dx	C77.x, C78.x, C79.x, C80.0
HIV disease	Dx	B20

Supplementary Table 3. Patient attrition

	**Overall**	
	**N**	**%**
**Number of patients in PHD who were aged 18 or older during 1/1/2017-3/31/2021**	93,251,105	
Patient has an inpatient visit OR 2 outpatient visits with an atrial fibrillation diagnosis	2,416,286	**100.0**
Patients from hospitals with continuous data from the 12-month look-back period to 12-month follow-up period	2,257,512	**93.4**
Patients who had continuous medical and pharmacy claims enrollment during the study period	76,217	**3.2**
Exclude patients with prior atrial fibrillation diagnosis	69,117	**2.9**
Exclude patients with prior oral anticoagulants use	40,392	**1.7**
Exclude patients with hip/knee replacement within 6 weeks prior to index	40,307	**1.7**
Exclude patients with non-qualifying diagnoses/procedures during look-back and/or index periods	23,148	**1.0**

Supplementary Table 4. Multivariable Adjusted Analysis Results for Assessing Time to initiate OAC and hospitalization during follow-up among OAC users, excluding Warfarin users

	**With OAC use**	**OAC initiated at index visit**	**OAC initiated during follow-up**	***p-value***
**Overall**	**9,307***	**6,970**	**2,337**	
All-cause hospitalization	2,981 (32.0%)	1,971 (28.3%)	1,010 (43.2%)	
Unadjusted OR	0.52 (0.47 - 0.57)			<0.001
Adjusted OR*	0.37 (0.33 - 0.42)			<0.001
Hospitalization due to stroke/SE	146 (1.6%)	84 (1.2%)	62 (2.7%)	
Unadjusted OR	0.45 (0.32 - 0.62)			<0.001
Adjusted OR*	0.33 (0.23 - 0.47)			<0.001

Abbreviations: OAC, oral anticoagulants; OR, odds ratio; SE, systemic embolism

*The total number of patients in each category.

**Adjusted for Age Category, Gender, Race, Ethnicity, Primary Payor, Care setting of index visit, Hospital Characteristics (Urban/rural, Geographic Region, Hospital Size, Teaching Status), Charlson Comorbidity Index, Obesity, Diabetes, Vascular disease history, and CHA_2DS_2-VASc score.

Supplementary Table 5. Multivariable Adjusted Analysis Results for Assessing Time to initiate OAC and hospitalization during follow-up among OAC users, by stroke history (including Warfarin users)_

	**With OAC use**	**OAC initiated at index visit**	**OAC initiated during follow-up**	***p-value***
**With stroke history**	**1,439***	**1,169**	**270**	
All-cause hospitalization	435 (21.8%)	317 (27.1%)	118 (43.7%)	
Unadjusted OR	0.48 (0.36 - 0.63)			<0.001
Adjusted OR**	0.44 (0.33 - 0.59)			<0.001
Hospitalization due to stroke/SE	61 (4.2%)	39 (3.3%)	22 (8.1%)	
Unadjusted OR	0.39 (0.23 - 0.67)			<0.001
Adjusted OR**	0.36 (0.20 - 0.65)			<0.001
**Without stroke history**	**9,620**	**7,116**	**2,504**	
All-cause hospitalization	3,260 (33.9%)	2,104 (29.6%)	1,156 (46.2%)	
Unadjusted OR	0.49 (0.45 - 0.54)			<0.001
Adjusted OR***	0.33 (0.30 - 0.37)			<0.001
Hospitalization due to stroke/SE	116 (1.2%)	65 (0.9%)	51 (2.0%)	
Unadjusted OR	0.44 (0.31 - 0.64)			<0.001
Adjusted OR***	0.32 (0.21 - 0.48)			<0.001

Abbreviations: OAC, oral anticoagulants; OR, odds ratio; SE, systemic embolism

*The total number of patients in each category.

**Adjusted for Age Category, Gender, Race, Ethnicity, Primary Payor, Care setting of index visit, Hospital Characteristics (Urban/rural, Geographic Region, Hospital Size, Teaching Status), Charlson Comorbidity Index, Obesity, Diabetes, Vascular disease history, and CHA_2DS_2-VASc score.

*** Adjusted for Age Category, Gender, Race, Ethnicity, Primary Payor, Hospital Characteristics (Urban/rural, Geographic Region, Hospital Size, Teaching Status), Charlson Comorbidity Index, Obesity, Diabetes, Vascular disease history, and CHA_2DS_2-VASc score.

Supplementary Table 6. Multivariable Adjusted Analysis Results for Assessing Time to initiate OAC and hospitalization during follow-up among OAC users, including additional covariates

	**With OAC use**	**OAC initiated at index visit**	**OAC initiated during follow-up**	***p-value***
**Overall**	**11,059***	**8,285**	**2,774**	
All-cause hospitalization	3,695 (33.4%)	2,421 (29.2%)	1,274 (45.9%)	
Adjusted OR**	0.33 (0.30 - 0.37)			<0.001
Hospitalization due to stroke/SE	177 (1.6%)	104 (1.3%)	73 (2.6%)	
Adjusted OR**	0.32 (0.23 - 0.44)			<0.001

Abbreviations: OAC, oral anticoagulants; OR, odds ratio

*The total number of patients in each category.

**Adjusted for Age Category, Gender, Race, Ethnicity, Primary Payor, Care setting of index visit, Hospital Characteristics (Urban/rural, Geographic Region, Hospital Size, Teaching Status), Charlson Comorbidity Index, Obesity, Diabetes, Vascular disease history, CHA_2DS_2-VASc score, Renal Disease, Dementia, Malignancy, Admission Type, and Discharge Status.

Supplementary Table 7. Multivariable Adjusted Analysis Results for Assessing Time to initiate OAC and hospitalization during follow-up among OAC users, among Patients with At Least One Refill

	**With OAC use**	**OAC initiated at index visit**	**OAC initiated during follow-up**	***p-value***
**Overall**	**8,094***	**6,238**	**1,856**	
All-cause hospitalization	3,695 (33.4%)	2,218 (35.6%)	930 (50.1%)	
Unadjusted OR	0.55 (0.49 - 0.61)			<0.001
Adjusted OR**	0.43 (0.38 - 0.48)			<0.001
Hospitalization due to stroke/SE	144 (1.8%)	88 (1.4%)	56 (3.0%)	
Unadjusted OR	0.46 (0.33 - 0.65)			<0.001
Adjusted OR**	0.34 (0.23 - 0.49)			<0.001

Abbreviations: OAC, oral anticoagulants; OR, odds ratio

*The total number of patients in each category.

**Adjusted for Age Category, Gender, Race, Ethnicity, Primary Payor, Care setting of index visit, Hospital Characteristics (Urban/rural, Geographic Region, Hospital Size, Teaching Status), Charlson Comorbidity Index, Obesity, Diabetes, Vascular disease history, CHA_2DS_2-VASc score.
